# Effects of early gestational parenteral iron administration on maternal iron biomarkers and reproductive performance in sows under commercial conditions

**DOI:** 10.1093/tas/txag141

**Published:** 2026-09-16

**Authors:** Katlyn McClellan, Darcy Pamulaklakin, Courtney Pohlen, Rebecca Swanson, Eric Weaver

**Affiliations:** Department of Animal Science, South Dakota State University, Brookings, SD 57007, United States; Department of Animal Science, South Dakota State University, Brookings, SD 57007, United States; Department of Animal Science, South Dakota State University, Brookings, SD 57007, United States; Department of Animal Science, South Dakota State University, Brookings, SD 57007, United States; Department of Animal Science, South Dakota State University, Brookings, SD 57007, United States

**Keywords:** sow, iron deficiency anemia, parenteral iron, iron dextran, inflammation, C-reactive protein

## Abstract

This study evaluated the effects of early-gestation parenteral iron dextran administration on maternal iron status and reproductive performance in sows under commercial conditions. A total of 235 TN70 sows (parities 1 to 9, average parity 4.2) were enrolled in early gestation (30 ± 2 days of gestation) and followed through pre-weaning (19 ± 2 days of lactation). Sows were allotted by parity and initial blood hemoglobin (Hb) to one of three parenteral iron dextran treatments initiated at enrollment: 0 mg (CONFe; *n* = 77), administered without injection; 2500 mg (MODFe; *n* = 79), administered as a single 12.5-mL intramuscular injection; or 5000 mg (HIGHFe; *n* = 79), administered as two 12.5-mL intramuscular injections (2500 mg per injection), with injections administered into the trapezius muscle. Blood Hb was measured on days 30 (enrollment) and 112 ± 2 days of gestation (pre-farrow) and day 19 ± 2 days of lactation (pre-weaning) using a HemoCue Hb 201 + system via ear-vein sampling. A subset of sows (*N* = 75; 25 per treatment) had jugular blood samples collected for analysis of serum iron, ferritin, total iron-binding capacity (TIBC), and plasma C-reactive protein (CRP). On day 112 of gestation, blood Hb and serum ferritin increased as iron dosage increased (linear, *P* = 0.016 and *P* = 0.046, respectively), whereas serum TIBC decreased (linear, *P* = 0.021). On day 19 of lactation, serum ferritin increased as iron dosage increased (linear, *P* = 0.036). Total born and liveborn piglets were not affected by treatment. However, stillborn rate tended to decrease as iron dosage increased (linear, *P* = 0.068). When evaluated within parity, stillborn rate decreased as iron dosage increased in parity 1 sows (linear, *P* = 0.024) and tended to decrease in sows of parity 5 or greater (linear, *P* = 0.097), whereas no responses were observed in intermediate parities. In conclusion, parenteral administration of iron dextran during early gestation improved sow iron status through weaning, with the greatest response generally observed at 5000 mg. Stillborn rate also tended to decrease as iron dosage increased, with responses most evident in parity 1 sows and sows of parity 5 or greater.

## Teaser

Early gestational parenteral iron supplementation improved sow iron status and reduced stillborn rate. These findings support further evaluation of injectable iron as a strategy to address iron deficiency in modern sows.

## Introduction

Anemia, typically defined as a whole-blood hemoglobin (Hb) concentration ≤ 10 g/dL, is common in modern sow herds, with a prevalence of approximately 50% reported across large-scale U.S. commercial systems (Castevens et al. 2020). Low Hb concentrations before farrowing have been associated with longer farrowing duration (McClellan et al. 2024b), greater stillborn rates, and increased sow removal (McClellan et al. 2026a), highlighting the importance of ensuring that sows enter the pre-farrow period with adequate iron status to support farrowing performance and continued productivity.

Increasing dietary iron supply alone may not improve maternal iron status because iron absorption and utilization are influenced by physiological status and the availability of other nutrients required for erythropoiesis, including amino acids, vitamins, and other trace minerals (Underwood and Suttle 1999; Koury and Ponka 2004; Goff 2018). Iron availability can be further influenced by systemic inflammation, which can reduce intestinal iron absorption and promote sequestration of iron within storage sites through hepcidin-mediated pathways (Schmidt 2015; Weiss et al. 2019). Consequently, inflammatory markers such as C-reactive protein (CRP), commonly evaluated alongside iron status in humans, can provide additional context when interpreting iron-related biomarkers and distinguishing iron deficiency from inflammation-associated changes in iron status (Richardson et al. 2025).

Given the complexity of dietary iron absorption and utilization, parenteral iron supplementation may provide a direct approach to supporting maternal iron status by bypassing the intestinal tract (Sharma et al. 2004). Previous research evaluating parenteral iron supplementation in sows has focused primarily on offspring outcomes, with limited attention given to maternal hematological responses (Pond et al. 1961). Of the limited research evaluating maternal responses, Bhattarai et al. (2019a) reported minimal changes in iron-related parameters following gestational iron supplementation in non-anemic sows. In contrast, more recent work has demonstrated that parenteral iron can effectively improve maternal iron status when administered during the post-weaning period. Intramuscular administration of 2500 or 5000 mg of iron dextran increased Hb and serum ferritin over a 30-d period, indicating enhanced erythropoiesis and improved iron stores (McClellan et al. 2026b). However, that study included a limited number of sows and was not designed to follow subsequent reproductive performance, leaving it unknown whether improvements in maternal iron status translate to reproductive benefits.

It remains unclear whether parenteral iron administered during gestation can improve maternal iron status before the substantial increase in maternal and fetal demands that occurs later in pregnancy. Furthermore, previous studies have not established an effective gestational dose or determined whether improving maternal iron status translates to measurable improvements in reproductive performance. Early-gestation administration provides a unique opportunity to increase maternal iron reserves well in advance of late gestation and farrowing, while allowing sufficient time for hematological responses to develop. Evaluating this approach under commercial conditions is particularly important given the high prevalence of anemia reported in modern sow herds and the associations between low Hb status and adverse reproductive outcomes. Therefore, the objective of this study was to evaluate the effects of increasing doses of early-gestation parenteral iron dextran on maternal iron status and reproductive performance of sows under commercial conditions.

## Materials and methods

### Animal care and use

All procedures were approved by the South Dakota State University Institutional Animal Care and Use Committee. Animals were housed and managed at a 2500-head commercial sow facility located in Winfred, South Dakota.

### Animals, housing, and diets

A total of 233 TN-line sows (parities 1 to 9; Avg parity: 4.2) were enrolled from early gestation (30 ± 2 days of gestation) through pre-weaning (19 ± 2 days of lactation). During gestation, sows were housed in individual stalls until approximately 1 week after pregnancy confirmation (45 days of gestation), after which they were moved to group pens until day 110 ± 2 of gestation. Sows were then transferred to individual farrowing crates until weaning. Throughout gestation, all sows were fed the farm’s standard gestation diet at 2.05 to 2.27 kg/d, depending on body condition. Upon entry into the farrowing facility, sows were fed the standard farm lactation diet at the same gestation feeding rate and were transitioned to ad libitum intake following farrowing. Both diets were formulated to meet or exceed NRC (2012) nutrient requirements and supplemented with ferrous sulfate monohydrate to provide approximately 112.5 mg/kg of iron (calculated).

### Iron dextran treatments

Sows were allotted by parity and initial Hb to one of three iron dextran (Uniferon; Pharmacosmos Inc., Watchung, NJ) treatments: 0 mg (CONFe; *n* = 77), 2500 mg (MODFe; *n* = 79), or 5000 mg (HIGHFe; *n* = 79).

### Iron injection administration

The CONFe sows received no injection. Sows in the MODFe group received a single 12.5-mL intramuscular injection (2500 mg) administered into the left trapezius muscle. Sows in the HIGHFe group received two 12.5-mL intramuscular injections (2500 mg per site), one in each trapezius muscle, to improve absorption and reduce localized irritation. All injections were administered slowly to minimize product fallout and ensure proper intramuscular deposition.

### Blood sampling

Blood Hb concentration was measured on days 30 ± 2 (enrollment) and 112 ± 2 (pre-farrow) of gestation and day 19 ± 2 (pre-weaning) of lactation using a point-of-care device. The ear vein was punctured using an 18-gauge × 1-inch needle, and a drop of blood was collected into a disposable microcuvette by capillary action. Microcuvettes were immediately analyzed using a HemoCue Hb 201 + device (HemoCue America, Brea, CA), which reports Hb concentration within 60 seconds. The HemoCue system has been validated for use in sows, with Hb measurements differing by less than 1% from laboratory measurements and remaining consistent between ear and jugular sampling sites (McClellan et al. 2025). At the same time points, approximately 10 mL of blood was also collected via jugular venipuncture from a subset of sows (*n* = 75; 25 per treatment), with 5 mL collected into a serum separator tube and 5 mL into an EDTA tube.

### Sow body condition assessment

Sow body condition was objectively assessed in all sows on days 30 ± 2 (enrollment) and 112 ± 2 (pre-farrow) of gestation and day 19 ± 2 (pre-weaning) of lactation using a sow body condition caliper, as described by Knauer and Baitinger (2017). The caliper was gently placed over the spine at the last rib to measure the angularity of the sow’s topline.

### Plasma analysis: acute phase protein

The EDTA blood samples were centrifuged at 2000 × *g* for 20 min. Plasma was harvested and stored at −20°C until analysis. C-reactive protein concentrations were determined using a commercially available Pig CRP enzyme-linked immunosorbent assay kit (Abcam, ab205089). Assays were performed according to manufacturer guidelines with modification to sample dilution to ensure values fell within the assay detection range. All reagents and samples were equilibrated to 18–25°C prior to analysis. Samples exceeding the standard curve were diluted and re-assayed. The assay detection range was 3.13 to 200 ng/mL, with intra- and inter-assay coefficients of variation <10%.

### Serum analysis: iron status markers

Serum separator tubes were centrifuged at 2000 × g for 20 min. Serum was harvested and stored at −20°C until analysis. Serum iron, serum ferritin, and total iron-binding capacity (TIBC) were quantified using the Vet AXCEL Clinical Chemistry System, according to the manufacturer’s instructions. Calibration and quality control procedures followed manufacturer specifications, and intra-assay variation was maintained below 10%.

### Reproductive performance

Reproductive performance records, including the number of totalborn, liveborn, stillborn, and mummified piglets, as well as sow removal data, were collected from the farm’s management software. Piglet fostering practices followed routine farm protocols to maintain normal production flow and could not be experimentally controlled. Therefore, the number of pigs weaned was not reported, as fostering practices may have confounded interpretation of this outcome.

### Sow removals

During the experimental period, 7 sows were removed from the CONFe group (5 abortions, 2 deaths), 2 sows were removed from the MODFe group (1 abortion, 1 death), and 5 sows were removed from the HIGHFe group (4 abortions, 1 death).

### Statistical analysis

All data were analyzed using SAS version 9.4 (SAS Institute Inc., Cary, NC). Assumptions of normality and homogeneity of variance were assessed before analysis. Continuous outcomes were analyzed using the MIXED procedure, with iron dextran treatment and parity as fixed effects. Treatment effects within parity were evaluated for blood Hb concentrations and reproductive performance only, with parity categorized as 1, 2, 3, 4, or ≥5. Treatment means were compared using Tukey’s adjustment, and orthogonal polynomial contrasts were used to evaluate linear and quadratic responses to increasing iron dextran dosage. Pearson correlations among iron-status biomarkers, CRP, and reproductive outcomes were evaluated using the CORR procedure. Associations between day 112 plasma CRP category (<30, 30 to 50, or >50 mg/dL) and reproductive outcomes were evaluated using the MIXED procedure. Anemia prevalence (Hb <10 g/dL; McClellan et al. 2024b) was evaluated using the FREQ procedure with a chi-square test. Significance was declared at *P* ≤ 0.05, and tendencies were noted at 0.05 < *P* ≤ 0.10.

## Results

### Sow blood hemoglobin and anemia prevalence

Blood Hb concentrations did not differ among treatments on day 30 of gestation, before iron administration (*P* = 0.927; Table 1). On day 112 of gestation, blood Hb concentrations were greater in HIGHFe sows than in CONFe sows, with MODFe sows being intermediate (*P* = 0.049), and Hb increased as iron dextran dosage increased (linear, *P* = 0.016). Blood Hb concentrations did not differ among treatments on day 19 of lactation. Changes in blood Hb concentrations between sampling time points did not differ among treatments; however, the change in Hb from day 30 to day 112 of gestation increased as iron dextran dosage increased (linear, *P* = 0.048). Anemia prevalence did not differ among treatments at any sampling time point (Fig. 1).

**Figure 1:**
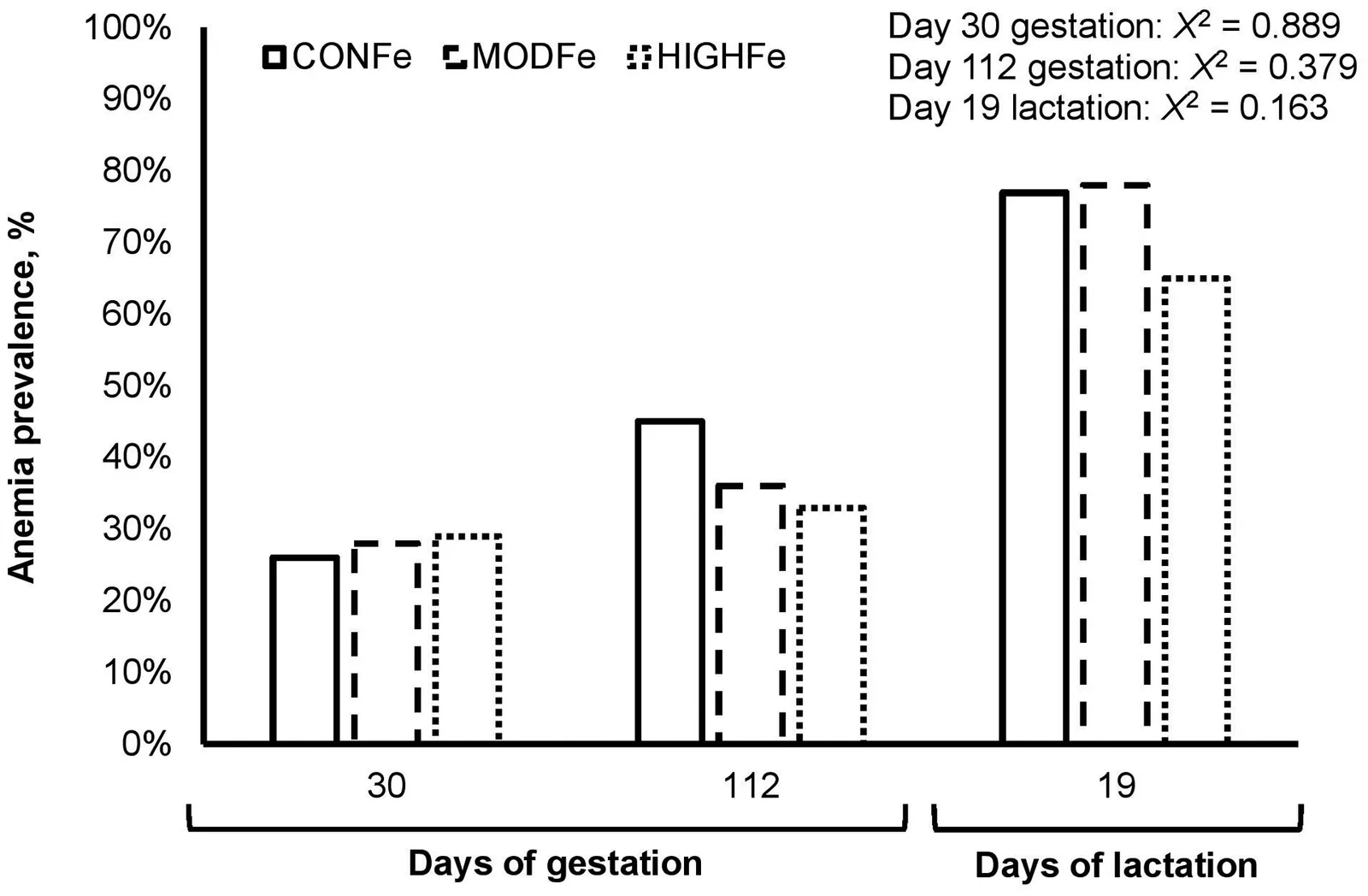
Anemia prevalence of sows administered intramuscular iron dextran at varying dosages in early gestation.^a–d^ ^a^CONFe, no iron dextran administered (0 mg); MODFe, 2500 mg iron dextran; HIGHFe, 5000 mg iron dextran administered intramuscularly at day 30 ± 2 of gestation. ^b^Day 30 of gestation–CONFe: 77 sows (average parity = 4.2); MODFe: 79 sows (average parity = 4.2); HIGHFe: 79 sows (average parity = 4.2). ^c^Day 112 of gestation–CONFe: 65 sows (average parity = 4.3); MODFe: 77 sows (average parity = 4.3); HIGHFe: 73 sows (average parity = 4.2). ^d^Day 19 of lactation–CONFe: 64 sows (average parity = 4.3); MODFe: 77 sows (average parity = 4.3); HIGHFe: 73 sows (average parity = 4.2).

**Table 1:** Blood hemoglobin (Hb) responses of sows administered intramuscular iron dextran at varying dosages in early gestation.

Item	Iron treatment^a^			SEM	*P-*value^b^		
	CONFe	MODFe	HIGHFe		Treatment	Linear	Quadratic
**Hb, g/dL**							
** Day 30 gestation^c^**	10.64	10.59	10.56	0.220	0.927	0.699	0.981
** Day 112 gestation^d^**	10.00^x^	10.17^xy^	10.46^y^	0.186	0.049	0.016	0.694
** Day 19 lactation^e^**	9.20	9.13	9.43	0.190	0.221	0.228	0.223
**Hb gestational change^f^**	−0.63	−0.40	−0.13	0.268	0.140	0.048	0.934
**Hb lactational change^g^**	−0.80	−1.04	−1.02	0.243	0.547	0.369	0.512
**Hb overall change^h^**	−1.43	−1.42	−1.13	0.282	0.450	0.276	0.546

a CONFe, no iron dextran administered (0 mg); MODFe, 2500 mg iron dextran; HIGHFe, 5000 mg iron dextran administered intramuscularly at day 30 ± 2 of gestation.

b Superscripts ^x,y^Represents comparisons made at *P* < 0.05.

c Day 30 of gestation–CONFe: 77 sows (average parity = 4.2); MODFe: 79 sows (average parity = 4.2); HIGHFe: 79 sows (average parity = 4.2).

d Day 112 of gestation–CONFe: 65 sows (average parity = 4.3); MODFe: 77 sows (average parity = 4.3); HIGHFe: 73 sows (average parity = 4.2).

e Day 19 of lactation–CONFe: 64 sows (average parity = 4.3); MODFe: 77 sows (average parity = 4.3); HIGHFe: 73 sows (average parity = 4.2).

f Differences refer to values on day 30 vs. day 112 of gestation.

g Differences refer to values on day 112 of gestation vs. day 19 lactation.

h Differences refer to values on day 30 of gestation vs. day 19 lactation.

When blood Hb responses were evaluated by treatment within parity, no treatment differences were observed in Hb concentrations at any sampling timepoint, changes in Hb between sampling timepoints, or anemia prevalence within any parity group (Table 2; anemia prevalence data not shown).

**Table 2:** Blood hemoglobin by parity in sows administered intramuscular iron dextran at varying dosages in early gestation.

Item	Iron treatment^a^			SEM	*P*-value		
	CONFe	MODFe	HIGHFe		Treatment	Linear	Quadratic
**Parity 1**							
**Number of sows, *n*^b^**	7	7	8	–	–	–	–
** Day 30 gestation^c^**	10.24	10.61	10.21	0.454	0.788	0.963	0.489
** Day 112 gestation^d^**	10.11	9.98	10.24	0.485	0.945	0.884	0.762
** Day 19 lactation^e^**	9.14	9.12	9.23	0.548	0.889	0.908	0.923
** Hb gestational change^f^**	−0.14	−0.64	0.03	0.433	0.574	0.835	0.304
** Hb lactational change^g^**	−0.97	−0.86	−1.01	0.466	0.980	0.988	0.850
** Hb overall change^h^**	−1.10	−1.49	−0.98	0.501	0.752	0.864	0.475
**Parity 2**							
**Number of sows, *n*^b^**	9	9	9	–	–	–	–
** Day 30 gestation^c^**	11.36	11.06	11.35	0.512	0.876	0.980	0.613
** Day 112 gestation^d^**	10.55	10.51	11.16	0.316	0.244	0.178	0.339
** Day 19 lactation^e^**	9.29	9.72	9.16	0.434	0.555	0.822	0.303
**Hb gestational change^f^**	−0.96	−0.44	−0.19	0.624	0.667	0.382	0.853
**Hb lactational change^g^**	−1.14	−0.79	−1.95	0.502	0.233	0.260	0.204
**Hb overall change^h^**	−2.05	−1.25	−2.10	0.715	0.624	0.966	0.339
**Parity 3**							
**Number of sows, *n*^b^**	18	20	19	–	–	–	–
** Day 30 gestation^c^**	10.80	10.47	10.58	0.256	0.643	0.553	0.468
** Day 112 gestation^d^**	9.76	9.98	10.28	0.270	0.404	0.183	0.906
** Day 19 lactation^e^**	8.91	8.92	9.10	0.257	0.843	0.608	0.782
**Hb gestational change^f^**	−1.01	−0.49	−0.32	0.321	0.283	0.130	0.620
**Hb lactational change^g^**	−0.86	−1.06	−1.20	0.328	0.758	0.460	0.941
**Hb overall change^h^**	−1.86	−1.54	−1.55	0.378	0.787	0.561	0.704
**Parity 4**							
**Number of sows, *n*^b^**	9	8	8	–	–	–	–
** Day 30 gestation^c^**	10.05	10.30	10.50	0.608	0.873	0.607	0.975
** Day 112 gestation^d^**	9.53	10.52	10.31	0.424	0.216	0.201	0.240
** Day 19 lactation^e^**	9.55	9.50	9.76	0.332	0.790	0.736	0.629
**Hb gestational change^f^**	−0.52	0.22	−0.34	0.557	0.598	0.824	0.331
**Hb lactational change^g^**	0.20	−1.30	−0.50	0.502	0.106	0.321	0.163
**Hb overall change^h^**	−0.32	−1.08	−0.85	0.588	0.645	0.538	0.496
**Parity ≥ 5**							
**Number of sows, *n*^b^**	27	33	30	–	–	–	–
** Day 30 gestation^c^**	10.52	10.33	10.48	0.224	0.818	0.906	0.534
** Day 112 gestation^d^**	10.00	10.22	10.46	0.183	0.196	0.072	0.967
** Day 19 lactation^e^**	9.26	9.18	9.73	0.176	0.151	0.158	0.125
**Hb gestational change^f^**	−0.52	−0.10	−0.06	0.243	0.317	0.172	0.501
**Hb lactational change^g^**	−0.73	−1.04	−0.72	0.237	0.523	0.972	0.256
**Hb overall change^h^**	−1.27	−1.15	−0.77	0.266	0.378	0.184	0.691

a CONFe, no iron dextran administered (0 mg); MODFe, 2500 mg iron dextran; HIGHFe, 5000 mg iron dextran administered intramuscularly at day 30 ± 2 of gestation.

b Prior to farrowing, 7 sows were removed from CONFe (average parity: 4.7), 2 sows were removed from MODFe (average parity: 1.5), and 5 sows were removed from HIGHFe (average parity: 5.2), and were therefore not included in farrowing performance outcomes.

c Day 30 of gestation–CONFe: 77 sows (average parity = 4.2); MODFe: 79 sows (average parity = 4.2); HIGHFe: 79 sows (average parity = 4.2).

d Day 112 of gestation–CONFe: 65 sows (average parity = 4.3); MODFe: 77 sows (average parity = 4.3); HIGHFe: 73 sows (average parity = 4.2).

e Day 19 of lactation–CONFe: 64 sows (average parity = 4.3); MODFe: 77 sows (average parity = 4.3); HIGHFe: 73 sows (average parity = 4.2).

f Differences refer to values on day 30 vs. day 112 of gestation.

g Differences refer to values on day 112 of gestation vs. day 19 lactation.

h Differences refer to values on day 30 of gestation vs. day 19 lactation.

### Sow iron biomarkers

Total serum iron concentrations did not differ among treatments at any timepoint (Table 3). Additionally, changes in total serum iron between sampling timepoints also did not differ by treatment.

**Table 3:** Serum iron, ferritin, and total iron binding capacity (TIBC) of sows administered intramuscular iron dextran at varying dosages in early gestation.

Item	Iron treatment^a^			SEM	*P*-value^b^		
	CONFe	MODFe	HIGHFe		Treatment	Linear	Quadratic
**Total iron, ug/dL**							
** Day 30 gestation^c^**	132.25	116.34	116.74	7.78	0.242	0.211	0.691
** Day 112 gestation^d^**	87.24	94.37	86.28	6.52	0.600	0.803	0.199
** Day 19 lactation^e^**	115.88	145.00	156.08	28.44	0.331	0.925	0.537
**Total iron gestational change^f^**	−42.23	−23.23	−29.32	9.52	0.304	0.291	0.202
**Total iron lactational change^g^**	35.00	20.12	34.67	8.70	0.351	0.980	0.062
**Total iron overall change^h^**	−7.89	−7.29	5.56	11.96	0.641	0.433	0.416
**TIBC, ug/dL**							
** Day 30 gestation^c^**	558.72	542.17	551.55	15.78	0.742	0.640	0.683
** Day 112 gestation^d^**	482.74^x^	460.61^xy^	454.37^y^	9.76	0.094	0.021	0.515
** Day 19 lactation^e^**	586.11	549.39	639.09	32.29	0.117	0.327	0.303
**TIBC gestational change^f^**	−75.96	−84.59	−91.05	17.00	0.811	0.410	0.826
**TIBC lactational change^g^**	87.00^y^	101.34^xy^	135.84^x^	17.03	0.101	0.041	0.611
**TIBC overall change^h^**	15.42	19.15	44.74	19.52	0.466	0.273	0.624
**Ferritin, ng/mL**							
** Day 30 gestation^c^**	14.62	14.68	14.52	0.84	0.989	0.984	0.815
** Day 112 gestation^d^**	14.57	17.92	18.29	1.44	0.126	0.046	0.402
** Day 19 lactation^e^**	17.20^b^^b^	22.78^a^^a^	24.03^a^^a^	1.56	0.005	0.036	0.256
**Ferritin gestational change^f^**	−0.53^y^	3.32^xy^	3.53^x^	1.47	0.087	0.031	0.320
**Ferritin lactational change^g^**	3.11	4.78	5.99	1.35	0.303	0.124	0.883
**Ferritin overall change^h^**	2.58^b^^b^	8.11^a^^a^	9.52^a^^a^	1.65	0.008	0.003	0.250

a CONFe, no iron dextran administered (0 mg); MODFe, 2500 mg iron dextran; HIGHFe,  5000 mg iron dextran administered intramuscularly at day 30 ± 2 of gestation.

b Superscripts ^aa,bb^ Represents comparisons made at *P* < 0.05 and ^x,y^ represents comparisons made at *P* < 0.10 across treatment.

c Day 30 of gestation–CONFe: 25 sows (average parity = 2.4); MODFe: 25 sows (average parity = 2.4); HIGHFe: 25 sows (average parity = 2.4).

d Day 112 of gestation–CONFe: 25 sows (average parity = 2.4); MODFe: 25 sows (average parity = 2.4); HIGHFe: 25 sows (average parity = 2.4).

e Day 19 of lactation–CONFe: 25 sows (average parity = 2.4); MODFe: 25 sows (average parity = 2.4); HIGHFe: 25 sows (average parity = 2.4).

f Differences refer to values on day 30 vs. day 112 of gestation.

g Differences refer to values on day 112 of gestation vs. day 19 lactation.

h Differences refer to values on day 30 of gestation vs. day 19 lactation.

Serum TIBC concentrations did not differ among treatments on day 30 of gestation before iron administration. On day 112 of gestation, HIGHFe sows tended to have lower serum TIBC concentrations than CONFe sows, with MODFe sows being intermediate (*P* = 0.094), and TIBC decreased as iron dextran dosage increased (linear, *P* = 0.021). Serum TIBC concentrations did not differ among treatments on day 19 of lactation. Changes in serum TIBC from day 30 to day 112 of gestation did not differ among treatments. From day 112 of gestation to day 19 of lactation, HIGHFe sows tended to have a greater increase in serum TIBC than CONFe sows, with MODFe sows being intermediate (*P* = 0.101), and the increase in TIBC was greater as iron dextran dosage increased (linear, *P* = 0.041). Overall changes in serum TIBC from day 30 of gestation to day 19 of lactation did not differ among treatments.

Serum ferritin concentrations did not differ among treatments on day 30 or day 112 of gestation. However, on day 112 of gestation, serum ferritin increased as iron dextran dosage increased (linear, *P* = 0.046). On day 19 of lactation, serum ferritin concentrations were greater in HIGHFe and MODFe sows than in CONFe sows (*P* = 0.005) and increased as iron dextran dosage increased (linear, *P* = 0.031). From day 30 to day 112 of gestation, HIGHFe sows tended to have a greater increase in serum ferritin than CONFe sows, with MODFe sows being intermediate (*P* = 0.087), and the increase in serum ferritin was greater as iron dextran dosage increased (linear, *P* = 0.031). Changes in serum ferritin from day 112 of gestation to day 19 of lactation did not differ among treatments. However, the overall increase in serum ferritin from day 30 of gestation to day 19 of lactation was greater in HIGHFe and MODFe sows than in CONFe sows (*P* = 0.008), and was greater as iron dextran dosage increased (linear, *P* = 0.003).

### Plasma C-reactive protein

Plasma CRP concentrations did not differ among treatments at any sampling time point (Table 4). Changes in CRP during gestation, lactation, or across the overall study period were not affected by iron dextran dosage.

**Table 4:** Plasma C-reactive protein (CRP) of sows administered intramuscular iron dextran at varying dosages in early gestation.

Item	Iron treatment^a^			SEM	*P-*value		
	CONFe	MODFe	HIGHFe		Treatment	Linear	Quadratic
**CRP, mg/dL**							
** Day 30 gestation^b^**	55.41	64.75	64.37	20.63	0.794	0.759	0.847
** Day 112 gestation^c^**	45.49	52.54	48.01	8.91	0.454	0.841	0.596
** Day 19 lactation^d^**	53.67	54.10	66.65	7.26	0.182	0.206	0.496
**CRP gestational change^e^**	1.30	−17.31	−16.48	23.78	0.690	0.597	0.739
**CRP lactational change^f^**	13.20	6.62	21.96	5.34	0.354	0.246	0.144
**CRP overall change^g^**	0.36	−13.97	−1.47	28.19	0.850	0.963	0.698

a CONFe, no iron dextran administered (0 mg); MODFe, 2500 mg iron dextran; HIGHFe, 5000 mg iron dextran administered intramuscularly at day 30 ± 2 of gestation.

b Day 30 of gestation–CONFe: 25 sows (average parity = 2.4); MODFe: 25 sows (average parity = 2.4); HIGHFe: 25 sows (average parity = 2.4).

c Day 112 of gestation–CONFe: 25 sows (average parity = 2.4); MODFe: 25 sows (average parity = 2.4); HIGHFe: 25 sows (average parity = 2.4).

d Day 19 of lactation–CONFe: 25 sows (average parity = 2.4); MODFe: 25 sows (average parity = 2.4); HIGHFe: 25 sows (average parity = 2.4).

e Differences refer to values on day 30 vs. day 112 of gestation.

f Differences refer to values on day 112 of gestation vs. day 19 lactation.

g Differences refer to values on day 30 of gestation vs. day 19 lactation.

Sows were categorized by CRP concentration on day 112 of gestation into three groups: <30 mg/dL (*n* = 16), 30 to 50 mg/dL (*n* = 25), and >50 mg/dL (*n* = 34) (Table 5). Parity did not differ among CRP classification, and total born and liveborn piglets were also not affected by CRP classification. However, sows with CRP concentrations >50 mg/dL had a greater stillborn percentage than sows in the <30 and 30 to 50 mg/dL categories (*P* = 0.013). In addition, stillborn percentage was positively correlated with CRP concentration on day 112 of gestation (*r* = 0.39; *P* = 0.004, data not shown). No correlations were observed between CRP concentrations and iron-related biomarkers, including Hb, ferritin, iron, and total iron-binding capacity, at any time point.

**Table 5:** Reproductive performance of sows categorized by average plasma C-reactive protein (CRP) concentration on day 112 of gestation.

Item	CRP ranges, mg/dL			SEM	*P-*value^a^
	<30	30–50	>50		
**Sows, *n***	16	25	34	–	–
**Parity, *n***	2.58	2.17	2.37	0.324	0.630
**Total born, *n***	16.17	16.94	17.79	1.007	0.392
**Liveborn, *n***	15.58	15.94	16.00	0.976	0.938
**Stillborn, %**	1.45^b^	3.18^b^	7.67^c^	1.815	0.013
**Mummified, %**	2.21	2.72	2.40	0.812	0.910

a Superscripts.

b,c Represents comparisons made at *P* < 0.05 across treatment.

### Sow body condition

Sow caliper scores did not differ among treatments at any sampling time point (Table 6). Changes in caliper scores between sampling time points also did not differ among treatments.

**Table 6:** Sow body condition responses to intramuscular iron dextran administered at varying dosages in early gestation.

Item	Iron treatment^a^			SEM	*P*-value		
	CONFe	MODFe	HIGHFe		Treatment	Linear	Quadratic
**Parity, *n***	3.1	3.1	3.1	–	–	–	–
**BCS, mm**							
** Day 30 gestation^b^**	14.83	14.56	14.43	0.349	0.509	0.255	0.823
** Day 112 gestation^c^**	15.41	15.10	15.23	0.381	0.718	0.639	0.497
** Day 19 lactation^d^**	14.61	14.09	14.59	0.385	0.290	0.940	0.116
**BCS gestational change^e^**	0.55	0.58	0.66	0.367	0.951	0.762	0.932
**BCS lactational change^f^**	−0.78	−1.01	−0.65	0.303	0.457	0.672	0.245
**BCS change^g^**	−0.22	−0.42	0.02	0.369	0.468	0.520	0.303

a CONFe, no iron dextran administered (0 mg); MODFe, 2500 mg iron dextran; HIGHFe, 5000 mg iron dextran administered intramuscularly at day 30 ± 2 of gestation.

b Day 30 of gestation–CONFe: 77 sows (average parity = 4.2); MODFe: 79 sows (average parity = 4.2); HIGHFe: 79 sows (average parity = 4.2).

c Day 112 of gestation–CONFe: 65 sows (average parity = 4.3); MODFe: 77 sows (average parity = 4.3); HIGHFe: 73 sows (average parity = 4.2).

d Day 19 of lactation–CONFe: 64 sows (average parity = 4.3); MODFe: 77 sows (average parity = 4.3); HIGHFe: 73 sows (average parity = 4.2).

e Differences refer to values on day 30 vs. day 112 of gestation.

f Differences refer to values on day 112 of gestation vs. day 19 lactation.

g Differences refer to values on day 30 of gestation vs. day 19 lactation.

### Reproductive performance

Total born and liveborn piglets did not differ among treatments (Table 7). However, stillborn rate tended to be lower in HIGHFe sows than in CONFe sows, with MODFe sows being intermediate (*P* = 0.066), and decreased as iron dextran dosage increased (linear, *P* = 0.068).

**Table 7:** Reproductive performance of sows administered intramuscular iron dextran at varying dosages in early gestation.

Item^a^	Iron treatment^b^			SEM	*P*-value^a^		
	CONFe	MODFe	HIGHFe		Treatment	Linear	Quadratic
**Number of sows, *n*^c^**	70	77	74	–	–	–	–
**Average parity, *n***	4.3	4.3	4.2	–	–	–	–
**Total born, *n***	17.77	17.68	17.56	0.457	0.948	0.681	0.976
**Live born, *n***	16.03	16.12	16.40	0.423	0.808	0.556	0.899
**Stillborn, %**	7.79^x^	6.05^xy^	5.21^y^	0.998	0.088	0.068	0.712
**Mummified, %**	2.00	2.77	1.40	0.674	0.472	0.403	0.380

a Superscripts ^x,y^ represents comparisons made at *P* < 0.10 across treatment.

b CONFe, no iron dextran administered (0 mg); MODFe, 2500 mg iron dextran; HIGHFe, 5000 mg iron dextran administered intramuscularly at day 30 ± 2 of gestation.

c Prior to farrowing, 7 sows were removed from CONFe (average parity: 4.7), 2 sows were removed from MODFe (average parity: 1.5), and 5 sows were removed from HIGHFe (average parity: 5.2), and were therefore not included in farrowing performance outcomes.

When reproductive performance was evaluated within parity (Table 8), stillborn rate in parity 1 sows tended to be lower in HIGHFe than in CONFe sows, with MODFe sows being intermediate (*P* = 0.057), and decreased as iron dosage increased (linear, *P* = 0.024). Reproductive performance did not differ among treatments in parity 2, 3, or 4 sows. Similarly, reproductive performance did not differ among treatments in sows of parity 5 or greater; however, stillborn rate tended to decrease as iron dosage increased (linear, *P* = 0.097).

**Table 8:** Reproductive performance by parity in sows administered intramuscular iron dextran at varying dosages in early gestation.

Item^a^	Iron treatment^b^			SEM	*P*-value^c^		
	CONFe	MODFe	HIGHFe		Treatment	Linear	Quadratic
**Parity 1**							
** Number of sows, *n***	7	7	8	–	–	–	–
** Total born, *n***	15.5	16.2	15.9	1.210	0.804	0.812	0.758
** Live born, *n***	13.8	15.0	15.2	1.455	0.967	0.222	0.410
** Stillborn, %**	10.84^x^	6.17^xy^	2.14^y^	2.535	0.057	0.024	0.926
** Mummified, %**	0.13	1.24	2.26	1.844	0.730	0.442	0.986
**Parity 2**							
** Number of sows, *n***	9	9	9	–	–	–	–
** Total born, *n***	17.57	16.13	16.83	1.052	0.563	0.609	0.365
** Live born, *n***	17.15	15.25	15.83	0.978	0.321	0.335	0.265
** Stillborn, %**	1.50	2.77	3.81	1.426	0.554	0.286	0.946
** Mummified, %**	0.75	2.25	1.91	1.375	0.679	0.544	0.551
**Parity 3**							
** Number of sows, *n***	18	20	19	–	–	–	–
** Total born, *n***	18.24	17.75	18.84	0.902	0.773	0.766	0.519
** Live born, *n***	17.01	16.65	17.73	0.802	0.629	0.549	0.460
** Stillborn, %**	4.56	4.08	4.84	1.24	0.928	0.889	0.718
** Mummified, %**	1.84	1.39	0.60	0.580	0.325	0.141	0.815
**Parity 4**							
** Number of sows, *n***	9	8	8	–	–	–	–
** Total born, *n***	16.89	18.00	19.25	1.442	0.599	0.443	0.685
** Live born, *n***	15.33	16.50	17.25	1.564	0.497	0.466	0.365
** Stillborn, %**	10.92	6.65	8.09	4.969	0.817	0.688	0.635
** Mummified, %**	1.91	2.08	2.63	1.460	0.506	0.888	0.341
**Parity ≥ 5**							
** Number of sows, *n***	27	33	30	–	–	–	–
** Total born, *n***	17.84	18.06	17.40	0.619	0.728	0.614	0.541
** Live born, *n***	15.58	16.13	16.02	0.580	0.770	0.592	0.628
** Stillborn, %**	10.32	7.35	6.67	1.547	0.207	0.097	0.530
** Mummified, %**	2.35	3.34	1.26	0.841	0.147	0.167	0.165

a Prior to farrowing, 7 sows were removed from CONFe (average parity: 4.7), 2 sows were removed from MODFe (average parity: 1.5), and 5 sows were removed from HIGHFe (average parity: 5.2), and were therefore not included in farrowing performance outcomes.

b CONFe, no iron dextran administered (0 mg); MODFe, 2500 mg iron dextran; HIGHFe, 5000 mg iron dextran administered intramuscularly at day 30 ± 2 of gestation.

c ^x,y^ Represents comparisons made at *P* < 0.10 across treatment within day.

## Discussion

The present study evaluated the effects of early gestational iron administration on maternal iron status and reproductive outcomes in commercial sows. This work addresses an important gap in understanding how injectable iron can be used to improve sow iron status before farrowing and whether these improvements translate into reproductive benefits under commercial conditions. Iron supplementation, particularly at the higher dose of 5000 mg, increased circulating Hb concentrations and improved other indicators of iron status through weaning. Stillborn rate also tended to decrease as iron dosage increased, suggesting that improving maternal iron status before farrowing may benefit reproductive performance.

The improvement in Hb near farrowing suggests that greater parenteral iron provision supported erythropoiesis during late gestation, a period characterized by increased maternal blood volume expansion and accelerated fetal micronutrient accretion (Matte and Girard 1996; Mahan et al. 2009; Peters and Mahan 2008). However, despite the relatively large quantity of iron administered, the absolute improvement in Hb was modest (<5%), and concentrations remained suboptimal. This suggests that iron availability may represent only one constraint on Hb synthesis in gestating sows and that increasing iron supply does not necessarily result in a proportional increase in circulating Hb.

Furthermore, treatment differences in Hb were no longer evident by pre-weaning. Previous work has demonstrated that Hb concentrations are often lowest around weaning (McClellan et al. 2024a), likely reflecting the cumulative physiological demands of late gestation and lactation. Because approximately 80 days elapsed between iron administration and the pre-farrowing assessment, the temporal Hb response following supplementation remains unknown. It is possible that Hb increased to a greater extent earlier following administration and subsequently declined as maternal and fetal iron demands increased through late gestation and lactation. Thus, although additional iron improved Hb during gestation, a single administration in early gestation produced only a modest improvement near farrowing and did not maintain greater circulating Hb concentrations through lactation. More frequent sampling following administration would be necessary to determine the timing and magnitude of the peak hematological response.

Although anemia prevalence was not influenced by iron supplementation, improvements in Hb with increasing iron provision suggest that categorical anemia thresholds may not fully capture biologically relevant changes in iron status. Animals may experience meaningful shifts in Hb while remaining on the same side of a diagnostic threshold. Previous work has demonstrated that a 1 g/dL increase in Hb was associated with an approximately 43% reduction in sow removal risk (McClellan et al. 2026a). Therefore, even modest improvements in Hb may have biological relevance, although the long-term implications of the magnitude of response observed herein remain to be determined.

Total serum iron concentrations were not influenced by iron supplementation. This is not unexpected, as serum iron reflects a transient pool of circulating iron that is tightly regulated and subject to substantial short-term fluctuation (Dale et al. 2002; Hoppe et al. 2003; Wei et al. 2008). As a result, serum iron is generally considered a relatively insensitive indicator of iron status unless animals are severely deficient. Consequently, the lack of response in serum iron does not necessarily indicate an absence of biological effect but rather highlights the limitations of this measure for assessing changes in iron status when homeostatic regulation of circulating iron is maintained.

The reduction in TIBC with increasing iron supplementation near farrowing provides additional evidence of improved maternal iron status. Total iron-binding capacity is inversely related to iron sufficiency and reflects the body’s capacity to transport additional iron, with greater values typically associated with increased iron demand (Kasvosve and Delanghe 2002). Therefore, lower TIBC with greater iron provision is consistent with reduced physiological demand for additional iron transport capacity. The subsequent increase in TIBC during lactation may reflect renewed iron demand as available iron was redistributed or utilized to support the physiological demands of lactation. Collectively, the Hb and TIBC responses indicate that increasing iron provision altered maternal iron metabolism through late gestation despite relatively modest changes in circulating Hb.

The ferritin response provides insight into the fate of iron administered during early gestation. Ferritin serves as the primary intracellular iron storage protein and is widely regarded as an indicator of body iron reserves (Knovich et al. 2009). The progressive improvement in ferritin with increasing iron provision, together with the persistence of greater ferritin through weaning, suggests that a portion of the administered iron was incorporated into maternal storage pools and remained available over an extended period. This may be particularly important given the substantial iron demands associated with rapid fetal growth during late gestation and the transition into lactation (Mahan 1990; Mahan et al. 2009). The ferritin response also provides important context for the relatively modest increase in Hb. Rather than all available iron being directed toward erythropoiesis, supplemental iron may have been partitioned among Hb synthesis, maternal iron storage, and other maternal or fetal iron-dependent processes. Therefore, the biological response to parenteral iron may be better represented by the collective improvement in iron-related biomarkers than by the magnitude of the Hb response alone. The persistence of greater iron stores through pre-weaning, despite the disappearance of treatment differences in Hb, further supports this interpretation.

Plasma CRP concentrations were not influenced by iron dextran administration, suggesting that parenteral iron did not elicit a measurable systemic inflammatory response at the sampling points evaluated. Additionally, despite the well-established relationship between inflammation and iron metabolism (Weiss et al. 2019), CRP was not associated with the iron-related biomarkers evaluated. Thus, the improvements in maternal iron status observed following supplementation do not appear to be readily explained by differences in systemic inflammatory status.

Previous work in 6- to 7-month-old pigs has demonstrated substantial differences in CRP concentrations between culled and clinically healthy animals (25 mg/dL vs. 8.5 mg/dL) (Chen et al. 2003). However, well-defined reference ranges for acute phase proteins in sows, including CRP, remain limited, particularly with respect to concentrations associated with reproductive performance. Although evaluating CRP classifications in relation to reproductive outcomes was not a primary objective of the present study, the limited information available in sows warranted further exploration of these relationships. Interestingly, greater CRP concentrations near farrowing were associated with increased stillbirth, suggesting that systemic inflammation during late gestation may be related to farrowing outcomes. This relationship may reflect compromised maternal health or physiological stress near parturition rather than a direct effect of CRP itself. Given the exploratory nature of this analysis and the lack of established CRP reference ranges in sows, these findings should be interpreted cautiously and should not be used to establish a biological threshold. Nevertheless, the observed relationship provides a unique basis for further investigation into the role of systemic inflammation in sow reproductive performance and whether CRP may serve as an indicator of reproductive risk.

Sow caliper scores were not influenced by treatment. Given that body condition is primarily driven by energy intake, nutrient partitioning, and overall dietary management (Monteiro et al. 2025), these findings suggest that iron supplementation alone is unlikely to influence sow condition in the absence of changes in energy or amino acid supply. Additionally, the lack of a treatment effect on body condition supports that differences in iron status were unlikely to be attributable to variation in sow condition across treatments.

Total born and liveborn piglets were not influenced by iron supplementation, which was expected given that treatment was administered after establishment of pregnancy. However, the reduction in stillbirth with increasing iron provision is consistent with previous work demonstrating relationships between maternal iron status near farrowing and reproductive outcomes. Reduced maternal Hb concentrations during late gestation have been associated with prolonged farrowing duration and increased stillbirth (McClellan et al. 2024b), suggesting that compromised iron status near parturition may negatively affect the sow’s ability to support the physiological demands of farrowing. This relationship is biologically plausible, as Hb is essential for oxygen transport, and reduced maternal oxygen-carrying capacity may increase susceptibility to fetal hypoxia during prolonged uterine contractions and delivery (Moore et al. 1956; Bhattarai et al. 2019b).

The effects of iron supplementation on stillbirth rate appeared to differ across parity, with the clearest dose-responsive improvement occurring in first-parity sows and evidence of a similar response in older sows (parity ≥ 5). These responses may represent two physiologically distinct periods of increased iron demand. First-parity sows are relatively young females that may still be undergoing maternal growth while also recovering nutrient reserves following their first reproductive cycle (Bikker and Blok 2017). This may be particularly relevant in the present herd, which historically experiences a pronounced parity 1 reduction in performance associated with inadequate lactation feed intake during the first reproductive cycle.

Conversely, iron status generally declines with advancing parity (McClellan et al. 2024a), potentially reflecting the cumulative demands of repeated gestation and lactation cycles. Older sows in this herd also historically produce larger litters, which may further increase maternal iron requirements as fetal number and total fetal mass increase. Thus, greater iron provision may benefit first-parity sows by supporting continued growth and recovery following their first reproductive cycle, whereas benefits in older sows may reflect accumulated depletion of iron reserves coupled with greater reproductive demands. However, the absence of a consistent response across intermediate parities and the smaller number of sows within individual parity classifications warrant caution when interpreting these relationships. Longitudinal research across successive reproductive cycles is needed to determine whether iron requirements and responses to supplementation differ with parity.

An important limitation of the present study was the extended interval between iron administration and subsequent assessment of iron status. The temporal response immediately following administration remains unknown, including when circulating Hb may have peaked and when administered iron was incorporated into storage pools. More frequent sampling following treatment would help characterize the partitioning of supplemental iron between erythropoiesis and storage and determine whether the modest late-gestation Hb response reflects a limited initial response or a decline from a greater response earlier in gestation. Such work is also needed to identify the optimal timing of supplementation within the reproductive cycle and determine how long improvements in iron status persist.

An additional limitation of parenteral iron as a strategy in sows is the limited understanding of tissue iron deposition and depletion following high-dose administration. The duration of iron retention in tissues and its potential implications for cull sow carcass quality and food safety remain unclear. These uncertainties have important implications for establishing practical supplementation strategies, including whether iron should be administered during every reproductive cycle or whether residual iron stores provide benefits across successive cycles, as well as determining appropriate timing relative to culling and potential withdrawal periods prior to marketing. Further research evaluating iron kinetics across successive reproductive cycles is warranted to establish the optimal timing, frequency, and long-term safety of parenteral iron supplementation in sows.

## Conclusion

In conclusion, early gestational administration of iron dextran improved maternal iron status, with responses increasing as iron dosage increased and the greatest overall response observed at 5000 mg. Stillborn rate also tended to decrease as iron dosage increased, with the lowest rate observed at 5000 mg and responses most evident in parity 1 and older sows. These findings indicate that parenteral iron may help sows achieve improved iron status before farrowing and support reproductive performance under commercial conditions. Further longitudinal research across multiple parities and reproductive cycles is needed to determine the optimal timing and frequency of administration. The safety of repeated high-dose administration, including the potential for tissue iron accumulation and other long-term effects, also requires further investigation.

## Funding

None declared.

## Conflicts of interest

None declared.

## List of abbreviations

CRP: C-reactive protein
Hb: hemoglobin
TIBC: total iron-binding capacity
